# Comparison of Models for Quantification of Tomato Brown Rugose Fruit Virus Based on a Bioassay Using a Local Lesion Host

**DOI:** 10.3390/plants11243443

**Published:** 2022-12-09

**Authors:** Shaheen Nourinejhad Zarghani, Mehran Monavari, Jens Ehlers, Joachim Hamacher, Carmen Büttner, Martina Bandte

**Affiliations:** 1 Division Phytomedicine, Faculty of Life Sciences, Albrecht Daniel Thaer-Institute of Agricultural and Horticultural Sciences, Humboldt-Universität zu Berlin, Lentzeallee 55-57, 14197 Berlin, Germany; 2 Section eScience, Federal Institute for Materials Research and Testing, Unter den Eichen 87, 12205 Berlin, Germany; 3 INRES-Plant Pathology, Universität Bonn, Nussallee 9, 53115 Bonn, Germany

**Keywords:** *Nicotiana* spp., infectiosity, virus concentration, modeling, mechanical inoculation

## Abstract

Considering the availability of serological and molecular biological methods, the bioassay has been paled into insignificance, although it is the only experimental method that can be used to demonstrate the infectivity of a virus. We compared goodness-of-fit and predictability power of five models for the quantification of tomato brown rugose fruit virus (ToBRFV) based on local lesion assays: the Kleczkowski model, Furumoto and Mickey models I and II, the Gokhale and Bald model (growth curve model), and the modified Poisson model. For this purpose, mechanical inoculations onto *Nicotiana tabacum* L. cv. Xanthi nc and *N. glutionosa* L. with defined virus concentrations were first performed with half-leaf randomization in a Latin square design. Subsequently, models were implemented using Python software and fitted to the number of local lesions. All models could fit to the data for quantifying ToBRFV based on local lesions, among which the modified Poisson model had the best prediction of virus concentration in spike samples based on local lesions, although data of individual indicator plants showed variations. More accurate modeling was obtained from the test plant *N. glutinosa* than from *N. tabacum* cv. Xanthi nc. The position of the half-leaves on the test plants had no significant effect on the number of local lesions.

## 1. Introduction

It is currently argued that *Tomato brown rugose fruit virus* (ToBRFV), a member of the virus genus *Tobamovirus*, in the family *Virgaviridae*, is of utmost concern for tomato production globally [1]. The virus caused an outbreak on tomato plants in Israel in 2014 [2]. ToBRFV has been reported from tomato (*Solanum lycopersicum* L.) [1,3,4,5,6,7,8,9,10,11,12,13,14,15,16,17,18,19], pepper (*Capsicum annuum* L.) [20,21,22,23], and weed hosts [24] in 35 countries [25], and has been causing devastating disease outbreaks in tomato production areas [26,27,28,29]. In fact, based on the Statistica report for 2020, tomato accounts for 16% of the global vegetable production [30] and is one of the most important vegetable crops in the world [31]. It has been reported that ToBRFV cause a yield loss of 15–55% [26]. The virus excites mild to severe mosaic symptoms with dark green bulges, leaf narrowing, and deformation. The peduncles and calyces often become necrotic and unable to produce fruit, and if fruits are produced, they will be infected with the virus, developing yellow blotches, brown or black spots, and rugose wrinkling. Such fruits are not marketable [25,32]. In addition, various plants are introduced as herbaceous hosts, such as *Nicotiana* spp. It has been reported that ToBRFV causes local lesions on *Nicotiana tabacum* L. cv. Xanthi nc and *N. glutionosa* L., as well as systemic infection in *N. benthamiana* Domin and *N. clevelandii* A. Gray [1]. In Israel, ToBRFV was detected in *Chenopodium murale* L. and *Solanum nigrum* L. around infected tomato crops [33].

Tobamoviruses including ToBRFV could be dispersed by the transport of contaminated seeds over a long distance. Moreover, they are stable viruses [34] and remain infectious for several years outside of the host cells on different surfaces and can serve as an inoculum source for later infections whenever a suitable host is available again [25,35,36,37,38]. Once tobamoviruses are introduced or established in the greenhouse, they spread quickly in the entire greenhouse during hands-on activity or pruning, as well as different surfaces of greenhouse tables, pots, and tools [39,40]. Understanding the epidemiology of ToBRFV is essential for managing the disease properly [39]. These include proper crop management and more stringent screening procedures at international borders using quick, accurate, and affordable diagnosis [41]. ToBRFV is a quarantine virus in the U.K. and some Latin American countries (Chile and Argentina), while in the EPPO region, it is locally present and is under official control [42]. In the EU, ToBRFV is a virus subjected to emergency measures (EU 2020/1191 and further implementations). Exclusion and eradication principles of plant disease management are applied to restrict its entrance and distribution and propagation materials must be tested for ToBRFV via defined methods. For instance, detection of ToBRFV on seeds is carried out in the EU by a standardized RT-PCR or real-time RT-PCR. In the EU, tomato fruit producers dealing with ToBRFV infection in their greenhouses must remove and destroy all infected plants from the production site, at least at the end of the cropping season. Additionally, they must apply specific hygiene measures on personnel, production site structures, machinery, and pruning tools to prevent ToBRFV spread (EU 2021/1809). The efficacy of various disinfectants to inactivate tobamoviruses such as ToBRFV has been tested in vitro using bioassays [40,43,44,45,46,47,48]. These assays map infectivity of the test suspensions, but do not allow quantification of viral load.

Virus quantification involves counting virus particles or viral genome fragment(s) in a known volume to determine their concentration. Applications are in diagnostics and in research, for example, in virus–host interaction studies. Several methods have been developed to determine the viral load in a given sample, based on chemical or physical principles. These methods include electron microscopy, serological assays such as ELISA (enzyme-linked immunosorbent assay), and q(RT)-PCR (quantitative reverse-transcribed polymerase chain reaction). None of these methods can show or confirm infectivity of the virus in plant sap or virus suspensions. Plant viruses are not viable themselves and depend on a living host. Therefore, only a bioassay can show infectivity of the virus and, if necessary, quantify infectious virus particles. However, molecular methods are more sensitive than bioassay. In 1929, Holmes established a local lesion assay and showed that the number of local lesions on the inoculated host depends on the virus titer [49]. Since then, a few models and equations have been developed, aiming to accurately correlate the number of local lesions on the indicator plant with the viral inoculum titer in the inoculum. These models can be categorized according to the underlying basic statistical equations. The models and their parameters are summarized in Table 1. The model of Bald in 1937 [50] is based on a Poisson series; those of Kleczkowski [51] and Furumoto and Mickey [52,53] are based on normal distribution and a confluent hypergeometric function, respectively. Furthermore, a ‘growth curve’ model or logistic model was presented by Gokhale and Bald [54], and finally, Bald [55] introduced a modified Poisson model (Table 1).

A biological assay is based on the viability or infectivity of a pathogen. In the case of plant viruses, a bioassay using a susceptible host plant is the only available method to verify pathogenicity. So far, the evaluation is mostly qualitative, i.e., the pathogen is infectious or non-infectious. A quantitative evaluation requires a reproducible relationship/regression to be established between the number of local lesions induced by the virus after artificial inoculation and the virus concentration in the inoculum.

In this study, we report (i) the accuracy of the different models for quantifying ToBRFV in virus suspensions and plant homogenates with a known virus concentration, and (ii) the suitability of the models for the prediction of virus concentrations in a plant homogenate with an unknown virus concentration.

## 2. Results

### 2.1. Propagation Hosts and Biotest Plants for ToBRFV Isolate DSMZ PV-1236

**Figure 1:**
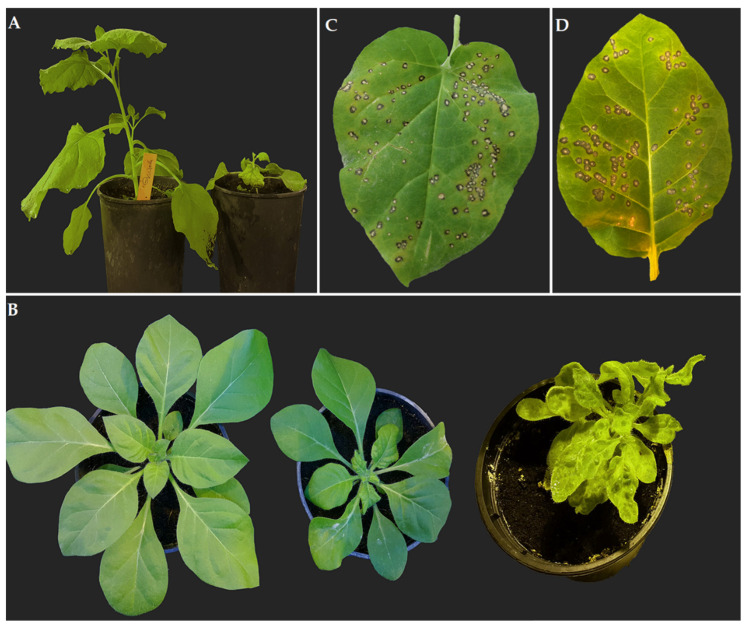
ToBRFV-induced symptoms on *Nicotiana* spp. after mechanical inoculation. (**A**) Severe stunting of *N. benthamiana* (left: mock-inoculated, right: ToBRFV-inoculated). (**B**) Left plant: mock-inoculated, Middle plant: early stunting and deformation of ToBRFV-inoculated *N. clevelandii,* Right plant: development of deformation and mosaic to the entire *N. clevelandii* plant. (**C**) Necrotic local lesions on *N. glutinosa* and (**D**) necrotic local lesions on *N. tabacum* cv. Xanthi nc.

Inoculated *N. benthamiana* plants at the 4–5 well-developed leaf stage with ToBRFV PV-1236 were systemically infected and showed stunting and severe leaf deformation in the newly emerged two top leaves 8–14 days post-inoculation (dpi). A few of the inoculated plants were kept for another 2 weeks, giving them more time to grow. These plants, however, stopped growing (Figure 1A). Lower leaves showed yellowing and necrotic veins. Three to four weeks post-inoculation (wpi), these plants died.

A similar scenario was documented when *N. benthamiana* plants were inoculated at the stage of 7–8 well-developed leaves, but the virus was detected as well in all non-inoculated leaves 10–14 dpi, giving the opportunity to have more infected plant material for virus purification. Since the protocol used for virus purification was based on a general tobamovirus purification receipt from inoculated *N. benthamina* plants, inoculation was repeated with enough plants to obtain at least 100 g of plant material for virus purification. The systemically infected leaves were collected 12 dpi. Systemic infection was established in *N. clevelandii* as well. The virus excited severe leaf deformation on newly developed leaves 10–14 dpi (Figure 1B), which extended to lower leaves in a way that 3–4 wpi, all leaves were deformed and sometimes showed narrowing. This deformation appears together with mosaic and yellowing of the entire shoot of the plant (Figure 1C). *N. clevelandii* was chosen as a source of inoculum for downstream experiments with the exception of virus purification. *N. clevelandii* plants were able to survive for at least two months more than *N. benthamiana* plants, making them a suitable candidate for keeping the inoculum alive in the greenhouse. In both *N. tabacum* cv. Xanthi nc and *N. glutionosa* plants, ToBRFV PV-1236 caused necrotic local lesions (Figure 1C,D). The virus was detected by DAS-ELISA in all inoculated plants of all four species. RT-PCR further confirmed infection of *N. benthamiana* and *N. clevelandii* with the inoculated isolate of ToBRFV by documentation of 195 bp DNA fragments on 1.2% agarose gel electrophoresis and sequence data.

### 2.2. Fitting of the Models to Bioassay Data

Inoculation of *N. glutinosa* with 100 µL of 10 and 5 mg/mL purified ToBRFV suspension caused severe damage of the leaves and necrosis in at least 30% of the inoculated half-leaves. Therefore, a maximum of 2 mg/mL of virus particles was used for inoculation experiments with the local lesion assay hosts *N. glutinosa* and *N. tabacum* cv. Xanthi nc to estimate parameters of the models and prediction of ToBRFV concentration in an unknown sample. The average number of the observed local lesions and those predicted by different models, as well as estimated parameters of each model are presented in Table 2 and Table 3 for *N. tabacum* cv. Xanthi nc and *N. glutinosa*, respectively. Since the numbers of local lesions (X) observed on different leaves after inoculation with the same virus preparation deviate greatly from a normal distribution, and the standard errors of x values depend on their magnitude, these data should be transformed [51].

**Figure 2:**
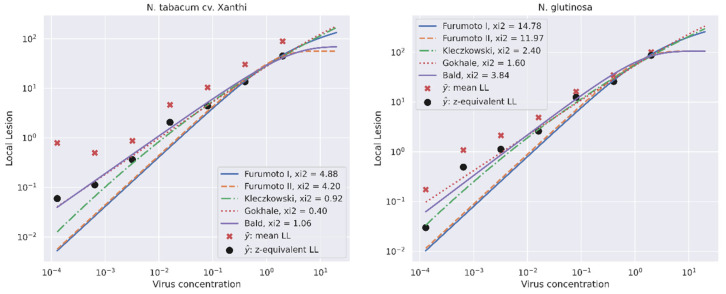
Mean number of the local lesions on *N. tabacum* cv. Xanthi nc (left) and *N. glutinosa* (right) against the virus concentrations in serially diluted ToBRFV suspensions with the dilution factor of 5 and starting concentration of 2 mg/mL plotted on log/log scale.

All five models were fitted to the local lesion number induced by ToBRFV suspensions with virus concentrations of 0.000128 up to 2 mg/mL in both local lesion hosts (Figure 2). The growth curve model and the Kleczkowski model had the best fit and the least *X^2^* error (Table 2 and Table 3). All models could fit the best with transformed means of the number of local lesions observed for inoculation of highest concentration of ToBRFV particles (i.e., 2 mg/mL) (Table 2). In the lower concentration range, a linear relationship was observed in the log/log scale between virus concentration and the number of local lesions, with the exception of the lowest concentration (0.000128 mg/mL), for which error dominates the data. When we extrapolate the maximum concentration of the virus by 10-fold, it is observed that the modified Poisson model and the Furumoto and Mickey model II convert to the horizontal asymptotes close to the highest $\hat{Y}$. The growth curve model and the Kleczkowski model predicted much higher maximum numbers of local lesions, as indicated by the model parameters N. The Furumoto and Mickey model I did not have an analytical limit. However, in our experimental data range, the Kleczkowski, growth curve, and modified Poisson models fit the data in both higher and lower ranges of ToBRFV concentration. Figure 2 shows the standard curves calculated by each model for serially diluted ToBRFV with a dilution factor of 5 and a starting concentration of 2 mg/mL plotted on a log/log scale.

### 2.3. Prediction of ToBRFV Concentration in a Spike Sample

The established fitted models were used to predict the concentration of ToBRFV in a spike sample using the estimated parameters shown for each local lesion host (Table 2 and Table 3). The virus concentration in the sample marked as unknown was estimated in the range of 0.37–0.53 and 0.88–1 mg/mL based on the different models on *N. glutinosa* and *N. tabacum* cv. Xanthi nc, respectively (Table 4). The best predictions were observed on *N. glutinosa* with the modified Poisson and Furumoto and Mickey II models, followed by the Kleczkowski model. Since the concentration of the sample was determined by UV spectroscopy as 0.4 mg/mL before inoculation, the computed concentration was close to the measured concentration when *N. glutinosa* was used as a local lesion host (0.37 and 0.47 mg/mL instead of 0.4 mg/mL). The application of a spike sample with a defined virus concentration enables the validation of the models in the prediction of the correct virus concentration in an unknown sample. Our results show that the host plant used in local lesion assays also has a significant effect on accuracy of the models to determine correct virus concentrations in virus inoculum.

### 2.4. Effects of Leaf Position and Local Lesion Host Species

According to the analysis of variance, the observed number of local lesions was statistically affected by the different local lesion hosts, *N. tabacum* cv. Xanthi nc and *N. glutinosa* (*p* value 0.01) (Table 5), regardless of the differences in leaf position. There were no statistically significant interaction(s) between the plant species, leaf position, and virus concentration.

## 3. Discussion

Although bioassays play a key role in several fields of plant virology, such as host range determination [57,58,59], symptomatology [60,61], virus replication, plant–pathogen interactions [62,63], and determination of function(s) of viral proteins or genes and host genes (gene knockdown via virus induced gene silencing) [64,65], the biological assay has always been paled into insignificance in virus quantification, because the sensitivity and reproducibility of this method might be misevaluated. Undoubtedly, molecular methods such as q(RT)-PCR or digital (RT)-PCR could detect very low concentrations or copy numbers of virus genomes [66,67], which is beyond the ability of a bioassay. The establishment of a successful bioassay or infectiosity test requires a minimum number of virus particles which is equal to the infective dose or the lowest values of boundary or the lowest concentration of virus particles that could be detected by this method. A similar phenomenon is known for plant pathogenic bacteria which is needed for attachment and expression of the pathogenic genes for the establishment of an infection [68] or the establishment of infection caused by fungi [69]. However, bioassays are the only methods to confirm infectiosity of plant pathogenic viruses, and neither qRT-PCR nor any other sensitive molecular method is able to detect the infectiosity of a virus suspension.

Local lesion assays must be carefully planned and conducted to minimize the variations and errors due to environmental conditions such as light cycle, temperature, species and age of local lesion host, and the position of leaves on the plant, as well as nutritional condition and methodology of inoculation. Keeping plants in darkness or low light intensity for 24 h prior to inoculation tends to increase the number of local lesions and will affect local lesion development. Any substances added during inoculation, such as phosphate-based inoculation buffers and particles of carborundum or diatomaceous earth, could positively influence local lesion formation [70]. Phosphate and abrasives will increase the number of local lesions [71], while any ribonuclease present will inhibit local lesion formation. Bentonite, which interferes with ribonuclease, will increase the number of local lesions produced as shown by naked TMV RNA, but not by intact virions [70]. It is suggested that if the source of inoculum is changed, for example, different processes of purification, the data obtained from randomized block designs may be subjected to analysis of variance (ANOVA) to determine the significance of differences between the number of lesions produced by different inocula, to make sure the inocula do not differ greatly from one another. Randomization is important, not just between leaves, but between plants in a treatment group. The results of this study showed that the position of the leaf or half-leaves was not statistically significant and did not affect the number of local lesions induced by ToBRFV in *N. tabacum* cv. Xanthi nc and *N. glutinosa*) (Table 5). However, the host plant played an important role in the quantification of ToBRFV. Therefore, *N. glutinosa* is deemed a better host than *N. tabacum* cv. Xanthi nc for the quantification of ToBRFV in bioassay. It has been shown that there were no significant differences in susceptibility between leaves in different positions when plants were grown under reduced light intensity [72,73]. The influence of the various factors mentioned above could be kept at bay by applying appropriate experimental designs and using statistical analysis in the evaluation of the data obtained [73]. When the number of inoculated half-leaf units from *N. glutinosa* plants exceeds 16, the accuracy of local lesion methods increases [73,74]. Therefore, using at least 24 half-leaf units for each dilution makes the accuracy of the method adequate in a way that statistically, there was no significant difference between the leaf positions.

The numbers of local lesions produced by different leaves when inoculated with the same virus preparation deviate significantly from a normal distribution, and the standard errors of x values depend on their magnitude. Therefore, before fitting the models, data should be appropriately transformed [56]. The author suggested the equation $z=\log\;\frac{1}{2}\;\left[x+c+\surd \left(x^{2}+2\mathrm{cx}\right)\right]$, where c is an arbitrary parameter. The parameter c could be optimized for each experiment. However, in this study, c = 20 offered the best transformation. Using this transformation is equivalent to using $\hat{Y}$, the z-equivalent of half-leaf count. To calculate $\hat{Y}$, first, the local lesion counts for a fixed concentration were transformed to normal distribution based on the above equation for z. $\hat{Y}$ is the inverse transformation of the mean of z-values. For more details, the readers are referred to [56].

Models for the correlation of the local lesion number to the virus concentration were developed as our understanding of virus–plant interactions increased. The Bald 1937 model is based on the Poisson distribution and it is assumed that a single virus particle can cause infection. Moreover, it is assumed that the leaf surface of the local lesion host can be infected with the same probability. Therefore, all the susceptible regions or entry points of a leaf have the same probability of infection with regard to the distribution of particles inside the regions, which follows the Poisson distribution. This model has two parameters, including the maximum number of local lesions observed in the assay and an unknown parameter which may account for the relative infectivity of any virus sample [50]. This model is also called the ‘one-particle-one-region’ model. The probability of causing all infections depends on the average number of virus particles inside the susceptible region. It is further assumed that this number is directly proportional to the plant virus concentration. This theory does not allow for any variation in the susceptibility between different susceptible regions of the host since it assigns the same probability for the entry points [51]. This model was not used in this study since it is a special case of the Furumoto and Mickey model.

Furumoto and Mickey [52,53] also followed the Bald 1937 model [50], the one-particle-one-region assumption. The main difference between these two models is that Furumoto and Mickey assumed that epidermal cells vary in their susceptibility to infection. There are a number of assumptions in the Furumoto and Mickey model: (i) virus particles, ***k*** in number, available outside a cell have a Poisson distribution with mean, α, proportional to concentration; (ii) the number of virus particles entering the cell with a fixed probability x of penetration has a binomial distribution with parameters ***k*** and x; and (iii) x has α, β distribution with parameters α and β and they arrive at the following model: Y = Nα(1+ cV/β). At that time, this hypermetric function was too complicated to use for fitting the data. Therefore, the authors simplified the modified equation to the formulae ‘model II’ as follows: Y/N=1$-{\;e}^{-\mathrm{cV}}$, which is the same model as described by Bald [50] with a different definition of N. Here, N is a hypothesized (not observed) number, which is usually estimated by inspection and extrapolation as the limit of lesions that appear at high concentrations of inoculum. Y/N is the expected proportion of infected regions in a total N of susceptible regions against the logarithm of virus concentration. Hence, Bald [50] can be assumed as a special case and was not investigated separately.

The Kleczkowski model [51] is based on the assumption that regional susceptibility (e.g., leaf surface) varies in such a way that the logarithm of minimal effective concentrations, necessary to cause the formation of the lesion, is normally distributed. In this model:

(1) $$Y=\frac{N}{\lambda \sqrt{2\pi }\;}\;\int_{-\infty }^{t}\exp\;\left\{-\frac{1}{2}\;{\left(\frac{t-\xi }{\lambda }\right)}^{2}\right\}\;\mathrm{dt}$$

where Y is the expected number of lesions per half-leaf, N is the mean number of ‘susceptible regions’ per half-leaf (which is a hypothesized number), t is the virus concentration in the inoculum or dilution of infective sap,$\;\xi ={\log\;x}_{0}$, $x_{0}$ = virus concentration or dilution of infective sap when 50% of the susceptible regions develop lesions, and λ is the standard deviation. This equation has three parameters, N, ξ, and λ, all of which are unknown and must be fitted to the experimental data.

Gokhale and Bald [54] derived the relationship between the expected proportion of infected regions measured by p = Y/N and the logarithm of concentration of virus (t = log x) as a growth curve model. In this model, they assumed that the rate of increase in p as a function of t is proportional to p and its deficiency (1-p). The resulting equation becomes:

(2) $$\frac{Y}{N}=\frac{1}{1+{\beta \;e}^{-\gamma t}}$$

where β and γ are positive constants of the model.

Bald et al. 1990 considered along the growth curve model a modified Poisson model resembling the Furumoto and Mickey model II. This modified Poisson model uses the Furumoto and Mickey model II to estimate C values for each observation, which in turn are used to estimate V and N. This model needs to be optimized. To this end, for each i, ${\tilde{C}}_{i}V_{i}$ is calculated from $Y_{\iota }=N\;(1-{\;e}^{-{\tilde{C}}_{i}V_{i}}$) $\left\{-{\tilde{C}}_{i}V_{i}=\;\ln\left(1-\frac{Y_{i}}{N}\right)\;\right\}$. In the next step, the log $\left({\tilde{C}}_{i}V_{i}\right)$ is regressed against the log $\left({\mathrm{CV}}_{\iota }\right)={\log\;m}_{i}$, logs of the observed concentrations, to obtain $\log\left({\tilde{C}}_{i}V_{i}\right)=a+b\;\log\;\left(m_{i}\right)$. These estimated parameters are used in the main equation

(3) $${\hat{Y}}_{ı}=\;N\;\left(1-e^{-{\mathrm{Am}}_{i}^{b}}\right)$$

where $A\;=10^{a}$.

When the developments of the models are compared, they share or differ in a few key basic assumptions which could be summarized as follows: (a) infection can start from a single virus particle that was introduced into a susceptible region of the host tissue; (b) each epidermal cell is a susceptible region; (c) cells differ in the probability of penetration by a virus particle; (d) all susceptible regions are identical; (e) a minimum dose of virus particles is needed to start an infection in a susceptible region; (f) the susceptibility of the regions varies in such a way that the logarithms of minimal infective doses are normally distributed. The Bald 1936 model follows assumptions a, b, and d; the Furumoto and Mickey models are based on assumptions a, b, and c; and the Kleczkowski model is based on assumptions e and f. In contrast, in the growth curve model, there were no clear explicit physical assumptions (e and f). It is a mainly data-based model and has been used accordingly for modeling disease development or for describing bacterial growth curves. The modified Poisson model is a modified version of the Poisson model to which, depending on the slope of the infection–dilution curve, it could be adopted. It is worth mentioning that the general performance of the presented models for ToBRFV is in accordance with the previously published data for TMV to which the models have been originally established [57]. This indicates that the underlying assumptions for these models could also be suitable for ToBRFV. Our results could not reject any of the models and their assumptions.

The application of a spike sample with a defined virus concentration enables the validation of the models in the prediction of the correct virus concentration in an unknown sample. Our results show that the host plant used in local lesion assays also has a significant effect on the accuracy of the models to determine correct virus concentrations in virus inocula. Since the underlying assumptions of these models about the susceptible regions are different, it is hard to infer which physical interpretation, such as susceptibility/tolerance of host or physical characteristics of the leaf surface, is the main reason for the higher accuracy of the model in *N. glutinosa* in comparison to *N. tabacum* cv. Xanthi nc. Based on the data presented in Table 4, it might be assumed that the production of more local lesions, especially in lower concentrations, might increase the accuracy of the prediction of virus concentration. In low concentrations, the growth curve, Kleczkowski, and modified Poisson models showed the best fit for high concentrations, and the modified Poisson model and Furumoto and Mickey model II converge to a maximum local lesion closer to the observed values. Therefore, we suggest using the modified Poisson model for predicting the concentrations of the ToBRFV in unknown samples.

Like other quantification methods such as qRT-PCR, for each new bioassay trial, a new dilution curve needs to be derived, because the slope of the dilution curve might not be constant among all individual experiments. As the average number of local lesions of each virus concentration in different trials might vary, this can affect the slope of the applied model. This observation was already made when the original models for TMV were developed [51].

It should be mentioned that the minimum number of virus particles that could cause a local lesion is still unknown. However, all the models can predict the minimum load of the virus needed for the production of a lesion. However, the interpretation of the minimum load depends on the assumption of the models. In the modified Poisson model and Furumoto and Mickey models I and II, it corresponds to the concentration of a virus suspension in which one particle can infect one cell (one-particle-one-region assumption). In the case of the Kleczkowski model, it corresponds to the distribution of a minimal dose of virus for the production of a lesion, and the growth curve model has no physical interpretation.

The inherent limitation of bioassay-based models is the need to observe at least one local lesion in any half-leaf unit to estimate the virus concentration. This means for any concentration that does not cause local lesions, models might underestimate the concentration as a negative control. This could be remedied by increasing the number of repetitions and decreasing the dilution factor from 10 or 5 to 2.

Therefore, in our experiment, we calculated two different virus concentrations: (a) the average of one lesion on all inoculated half-leaf units and (b) the observation of only one lesion in a total of 24 half-leaf units. In the case of ToBRFV on *N. glutinosa* in this study, based on the Kleczkowski model, they were calculated as 0.00321 mg/mL and 8.37 × 10^−6^ mg/mL for items a and b, respectively. It should be mentioned that real-time RT-PCR could detect a lower concentration of the virus, and it is more sensitive than a bioassay; therefore, based on plant health regulations, RT-PCR and real-time RT-PCR are recommended for the detection of the virus.

We showed the capability of the presented models based on bioassays in the estimation of the virus concentration in an unknown sample after fitting the models on serially diluted virus particles with a defined concentration (standard curve based on local lesion numbers). Thus, there is opportunity to quantify the virus concentration. In the case of disinfection treatments or any physical treatment (such as thermotherapy), the reduction in the virus titer can be measured, and subsequently, the efficacy of the treatment can be calculated. Based on our knowledge, it is the first report of the quantification of ToBRFV based on bioassay and it offers new insights through the assessment of viral load. In addition, the presented analysis and assessment of bioassay data could also be used in other fields of application where quantitative evaluation is required but chemicals/ingredients hamper RT-PCR, such as screening for antiviral agents, polymers, and virucides. For example, quantitative changes in virus titers during disinfection treatments on seeds and even on different surfaces in greenhouses can be determined. Thus, the efficiency of those treatments can be ascertained in detail, and weak points caused by the process or the user can be identified and used to develop new control strategies.

## 4. Materials and Methods

### 4.1. Virus Source, Systemic and Local Lesions Hosts

ToBRFV isolate PV-1236 was obtained from DSMZ (German Collection of Microorganisms and Cell Cultures GmbH, Braunschweig, Germany) and was propagated on *N. benthamiana* as a systemic host when they had 4–5 and 7–8 well-developed leaves. In both cases, the systemic leaves were collected 10–14 dpi and were kept at ∗70 °C until they were subjected to virus purification. As a second systemic host, *N. clevelandii* was used as a source of inoculum for bioassays. Systemically infected *N. clevelandii* leaves were inoculated when they had 7–8 well-developed leaves to obtain more infected plant material for downstream inoculation assays. Theses leaves were collected four weeks after inoculation of *N. clevelandii*, mixed and homogenized with mortar and pestle with the help of liquid nitrogen. The ground leaves were immediately stored at −70 °C until they were used as a source of inoculum in some serial dilution–infection experiments.

In general, three types of inocula were used in bioassays: (i) infected *N. clevelandii* leaves, (ii) purified particles, and (iii) purified particles mixed with sap of a non-infected *N. clevelandii*. The latter, a so-called spike sample, contained a defined number of ToBRFV particles and was used to determine the accuracy of the models in calculating the virus concentration.

Bioassays were comparatively carried out with two local lesion hosts recommended for tobamoviruses: *N. tabacum* cv. Xanthi nc and *N. glutinosa* [57,75]. The latter served as a local lesion host in establishing the models to correlate virus concentration and observed local lesions induced by tobacco mosaic virus. Furthermore, that plant species was recommended as a local lesion host for tobamoviruses for bioassay [76]. The *Nicotiana* spp. plants were grown in pots filled with bedding substrate (Klasmann-Deilmann GmbH, Geeste, Germany) at 25 °C, and a photoperiod of 16 h light and 8 h dark.

### 4.2. Mechanical Inoculation and Experimental Design of Local Lesion Bioassays

The purified ToBRFV particles were serially diluted in distilled water with dilution factors of 5 or 10. Then, 100 µL of each dilution was inoculated on each half-leaf. Just before inoculation, Celite was added to all dilutions to a final concentration of 3% (*w/v*). To prepare spike samples, *N. clevelandii* leaves ground in the presence of distilled water in a 1:5 ratio and purified virus particles were added to the first dilution (1:5) to a final concentration of 0.4 mg/mL. The number of local lesions was recorded 5–7 dpi. This spike sample was used as a treatment in the same experiments with dilution series.

Inoculations on test plants were performed as the half-leaf method and randomized [51,76] in Latin square design. A total of 24 (8 × 3) half-leaf units were inoculated for each dilution. All experiments were repeated at least three times. The effects of leaf position and host plant on the number of local lesions were analyzed in a factorial Latin square design.

### 4.3. Purification of ToBRFV

A total of 100 g of ToBRFV infected *N. benthamiana* leaves was homogenized in 300 mL 0.1 M phosphate buffer (pH = 7) containing 0.1% (*w/v*) Na-DIECA and 0.1% (*v/v*) thioglycolate with a Waring blender and squeezed through two layers of cheesecloth. Then, 150 mL chloroform was added to the filtrate and mixed on a stirrer for 10 min. The resulting emulsion was clarified by low-speed centrifugation for 20 min at 5000× *g* in a swinging bucket rotor. The supernatant was concentrated by ultracentrifugation for 90 min at 90,000× *g* with a fixed-angle rotor using steel beakers. The supernatant was discarded and the resulting pellets were resuspended in 5 mL of phosphate buffer (0.1 M pH = 8) containing 0.01 M ascorbic acid and centrifuged at 5000× *g* at 4 °C for 10 min. The supernatant was ultracentrifuged through 20 mL of a 30% sucrose cushion per polycarbonate bottle for 2.5 h at 90,000× *g* in a Beckman L7.55 ultracentrifuge and a Ti 45 titanium fixed-angle rotor. The sediments were resuspended in 2 mL of 0.1 M phosphate buffer per tube and homogenized with a glass potter. The resulting homogenate was centrifuged at 12,000× *g* for 15 min. The supernatant was centrifuged in CsCl solution (1.325 g/cm^3^) at 90,000× *g* overnight in a vertical rotor. The ToBRFV bands were removed with a syringe, diluted with two volumes of phosphate buffer (0.01 M pH = 8), and submitted to a second CsCl density gradient to obtain a pure virus suspension. The subtracted ToBRFV bands were mixed with 0.1 M phosphate buffer pH 7.0 and again centrifuged for 4 h in the fixed-angle rotor at 90,000× *g*. The sediment of purified virus particles was resuspended in 0.1 M phosphate buffer (pH = 7) containing 0.01 M Na-EDTA and kept at 4 °C for further usage.

### 4.4. Spectrophotometry

The virus concentration in the virus suspension was measured via spectrophotometry with two different devices: BioPhotometer 6131 (Eppendorf, Hamburg, Germany) using a macro cuvette (1 mL volume) and -UV/VIS-Spectralphotometer NanoDrop™ One/OneC (Thermo Scientific™, Madison, WI, USA). Each sample was measured at least 5 times at 260 nm, and the mean value was used to calculate the virus concentration based on the Lambert–Beers equation. A specific extinction coefficient of E_260_ nm 0.1% = 3.18 determined for TMV (Zaitlin 2000) was used for calculation of the virus concentration of the purified ToBRFV particles.

### 4.5. Correlation of Local Lesion with ToBRFV Concentration and Statistical Analyses

Data transformation was applied using the equation $z=\log\;\frac{1}{2}\;\left[x+c+\surd \left(x^{2}+2cx\right)\right]$, where x is the lesion number and c is a constant obtained by plotting standard deviations against the means and extrapolating the regression line to the abscissa [56]. The Python SciPy library was used for implementing the models and fitting the data. The Kleczkowski (1950), Furumoto and Mickey models I and II [52,53], and growth curve [54] models were fitted to virus concentrations and the corresponding number of local lesions. To this end, we used the weighted least square method and minimization through the ‘trf’ Trust Region Reflective algorithm. In fitting the models, the square errors were weighted inversely with the expected number of lesions $\hat{Y_{i}}$:

(4) $$\chi^{2}=\sum_{i=1}^{n}\frac{{\left(Y_{i}-\hat{Y_{i}}\right)}^{2}}{\hat{Y_{i}}}$$

Due to uncertainty in the experiments, we assume that the standard deviation of errors in Y data is $\sigma_{y}=\sqrt{\hat{y}}$.

The Kolmogorov–Smirnov test was used to check the normality of the data. Analysis of variance (ANOVA) was used to determine the statistical effect of plant species and leaf position in observed local lesions. Differences between means were assessed using the Tukey HSD test at *p* < 0.05. Statistical analyses were performed with SPSS version 25 (IBM, Chicago, IL, USA).

### 4.6. Detection of ToBRFV

To confirm the ToBRFV origin of symptoms observed on systemic and local lesion hosts, appropriate leaves were subjected to DAS-ELISA (double antibody sandwich ELISA). The serological tests were applied using the ToBRFV-specific antibody and conjugate RT-1236 following the supplier’s instructions (DSMZ, Braunschweig, Germany). To perform RT-PCR, total RNA was extracted from leaves using the Spectrum™ Plant Total RNA Kit (Sigma-Aldrich, Taufkirchen, Germany), and 1.5 µg of the isolated RNA was subjected to cDNA synthesis in a 20 µL reaction volume containing 100 pmol random hexamers, 1× transcriptase buffer, 2 mM dNTPs, 20 units of RNasin, and 100 units of Maxima H minus reverse transcriptase (Thermo Scientific™, Madison, WI, USA), following the thermal profile recommended by the manufacturer (Thermo Scientific™). The Phusion™ High-Fidelity DNA Polymerase kit (Thermo Scientific™) was used for a 20 µL PCR reaction containing 1 µL of synthesized cDNA as a template, and the primer pair of ToBRFV-1482-s (5′-TAATCAGCAAGTTTAGTTTG-3′)/ToBRFV-1677-as (5′-TCAGTCACTAATCTATCGTG-3′) following the supplier’s instructions. The primers were designed in this study with the help of Primer Express™ Software v3 (Applied Biosystems, Foster City, CA, USA). The RT-PCR products were sent for direct sequencing from both directions with PCR product-specific primers (Macrogen Europe, Amsterdam, The Netherlands).

## Figures and Tables

**Table 1:** Overview of different models used for correlation of virus concentration in the inoculum and number of local lesions produced after inoculation. The equation and an explanation of the parameters used are given in each case.

Name of the Model	Hypothesis/Assumption	Equation	Model Parameters
Bald 1937 model [50] *	(i) one-particle-one-region (ii) same susceptibility of regions	$y=N\;(1-{\;e}^{-\mathrm{ax}}$)	a
Kleczkowski model [51] **	(i) minimal dose of virus is needed (ii) variable susceptibility of regions	$Y=\frac{N}{\lambda \sqrt{2\pi }\;}\;\int_{-\infty }^{t}\exp\;\left\{-\frac{1}{2}\;{\left(\frac{t-\xi }{\lambda }\right)}^{2}\right\}\;\mathrm{dt}$	*N, ξ, λ*
Furumoto and Mickey model I [52,53] ***	(i) one-particle-one-region (ii) each cell is a susceptible region with max. of 400,000 (iii) susceptibility of the regions follows beta distribution	Y = Nα ln(1+ cV/β)	Nα and c/β
Furumoto and Mickey model II [52,53] ***	(i) one-particle-one-region (ii) each cell is a susceptible region (iii) follows beta distribution	Y = N (1$-{\;e}^{-\mathrm{cV}}$) (model II)	N and c
Gokhale and Bald model or Growth curve model [54] ****	(i) no explicit physical assumption (ii) growth curve relation between expected proportion of infected regions and the log of virus concentration	$Y\left(t\right)=\frac{N}{1+{\beta e}^{-\gamma t}}$	N, β and γ
Bald et al., 1990 model or Modified Poisson model [55] *****	modified Poisson model	${\hat{Y}}_{ı}=\;N\;\left(1-e^{-{\mathrm{Am}}_{i}^{b}}\right)$ A = 10^a^	A, m, b

* where y is the number of lesions produced at a relative concentration *x* of the virus suspension; N is the maximum number of lesions obtainable. The parameter *a* includes *pn_1_*, where p is the probability of a single particle entering to cause infection, n is the number of possibly infective particles, and n_1_ is the value of n for an undiluted sample of inoculum. ** Y is the expected number of lesions per half-leaf, N is the mean number of ‘susceptible regions’ per half-leaf, which is a hypothetical number, t=x (x is virus concentration in the inoculum or dilution of infective sap), $\xi ={\log\;x}_{0}\;$($x_{0}\;$= virus concentration or dilution of infective sap when 50% of the susceptible regions develop lesions), and λ is the standard deviation. *** Y is the expected number of lesions per half-leaf, N is the total number of susceptible regions; *c* is the constant V is the virus concentration in the inoculum. α and β are constants determining the distribution of cell penetration probabilities. This equation has two parameters, the values of which are unknown and must be adjusted to give the best possible fit to the experimental data. It is evident that the parameters N and α as well as c and β are not identifiable separately. **** Y is the expected number of lesions out of a total possible number N, t = log x, x being the virus concentration in the inoculum, β and γ positive constants. ***** The modified Poisson model uses Furumoto and Mickey model II to estimate c values for each observation, which in turn are used to estimate V and N. This model needs to be optimized. To this end, for each i, ${\tilde{C}}_{i}V_{i}$ is calculated from $Y_{i}=N\;(1-{\;e}^{-{\tilde{C}}_{i}V_{i}}$) $\left\{-{\tilde{C}}_{i}V_{i}=\;\ln\;\left(1-\frac{Y_{i}}{N}\right)\right\}$. In the next step, the log $\left({\tilde{C}}_{i}V_{i}\right)$ is regressed against the log $\left({\mathrm{CV}}_{i}\right)={\;\log\;m}_{i}$, logs of the observed concentrations, to obtain log $\left({\tilde{C}}_{i}V_{i}\right)=a+b\;\log\;\left(m_{ı}\right)$. These estimated parameters are used in the main equation.

**Table 2:** Mean number of observed necrotic local lesions on *N. tabacum* cv. Xanthi nc with serially diluted ToBRFV suspensions and related means of transformed data and computed data based on the different models. Data transformation performed according to Section 4.5.

Virus Concentration (mg/mL)	Mean No. of Observed LL *	$\hat{Y}$ **	Computed $\hat{Y}$ Based on				
			Kleczkowski Model	Furumoto and Mickey Model I	Furumoto and Mickey Model II	Growth Curve Model	Modified Poisson Model
2	89.58	45.24	44.07	46.94	44.84	44.65	41.333
0.4	30.45	13.69	15.08	14.06	15.25	14.49	16.169
0.08	10.45	4.43	4.61	3.18	3.44	4.53	5.190
0.016	4.66	2.07	1.26	0.65	0.71	1.40	1.563
0.0032	0.87	0.37	0.30	0.13	0.14	0.43	0.462
0.00064	0.5	0.11	0.07	0.03	0.03	0.13	0.136
0.000128	0.79	0.06	0.01	0.01	0.01	0.04	0.040
Model parameters ***			N: 9266.07	N: 45.24	N: 56.65	N: 581.86	N: 69.44
			λ: 2.00	C: 0.91	c/β: 0.78	β: 20	c: 0.78
			ξ: 5.49			γ: 1.69	A: 0.64
							b: 0.76
Error *X^2^*			0.53	4.88	4.20	0.4	1.06

* local lesion. ** $\hat{Y}$ is the z-equivalent of half-leaf count. First, each local lesion count for a fixed concentration was transformed to normal distribution. $\hat{Y}$ is the inverse transformation of the mean of z values and used for fitting the data. *** The model parameters: N, λ, ξ, C, c/β, β, γ, A, b, and c are explained in Table 1.

**Table 3:** Mean number of observed necrotic local lesions on *N. glutinosa* with serially diluted ToBRFV suspensions and related transformed and computed based on the different models. Data transformation performed according to Section 4.5.

Virus Concentration (mg/mL)	Mean of Observed No. LL	$\hat{Y}$ *	Computed $\hat{Y}$ Based on				
			Kleczkowski Model	Furumoto and Mickey Model I	Furumoto and Mickey Model II	Growth Curve Model	Modified Poisson Model
2	101.59	87.13	84.09	91.12	85.95	85.79	83.78
0.4	34.86	25.91	30.35	27.37	30.32	28.84	36.58
0.08	16.21	12.48	9.79	6.21	6.92	9.36	11.39
0.016	4.91	2.62	2.82	1.28	1.42	3.00	3.19
0.0032	2.13	1.13	0.72	0.26	0.29	0.96	0.86
0.00064	1.09	0.49	0.16	0.05	0.06	0.31	0.23
0.000128	0.17	0.03	0.03	0.01	0.01	0.10	0.06
Model parameters **			N: 11,064.25	N: 87.13	N: 105.14	N: 1134.53	N: 105.14
			λ: 2.00	C: 0.92	c/β: 0.85	β: 20	A: 1.03
			ξ: 5.16			γ: 1.64	b: 0.82
							c: 0.82
Error *X^2^*			2.4	14.78	11.97	1.60	3.84

* For explanation, see Table 2. ** The model parameters are explained in Table 1 and Table 2.

**Table 4:** Predicted concentration of a spike sample with 0.4 mg/mL ToBRFV based on different models.

Host	$\hat{Y}$	Kleczkowski Model	Furumoto and Mickey Model I	Furumoto and Mickey Model II	Growth Curve Model	Modified Poisson Model
*N. tabacum* cv. Xanthi nc	28.302	1.008 mg/mL	0.954 mg/mL	0.883 mg/mL	1.030 mg/mL	0.977 mg/mL
*N. glutinosa*	34.867	0.49 mg/mL	0.533 mg/mL	0.473 mg/mL	0.5266/mg/mL	0.372 mg/mL

**Table 5:** ANOVA test results for evaluation of effects of leaf position and type of local lesion host on the number of local lesions.

Source	df *	Sum of Squares	*F* Value	Pr > *F*
Treatment	47	40.72	11.01	<0.0001
Host species (a)	1	0.93	11.83	0.0007
Position of the leaf (b)	2	0.24	1.55	0.1890
Virus concentration (c)	7	36.38	66.03	<0.0001
a × b	2	0.03	0.19	0.8252
a × c	7	0.69	1.25	0.27
b × c	14	1.44	1.31	0.20
a × b × c	14	1.00	0.91	0.54
Error	336	26.45		
Corrected Total	383	67.17		

* df: degree of freedom. a: plant species (*N. tabacum* cv. Xanthi nc and *N. glutinosa*). b: position of the leaf, the top three well-developed leaves (L1, L2, L3). c: virus concentration in 8 preparations serially diluted with the dilution factor of 5, starting with 2 mg/mL. Data were transformed using the following equation: $z=\log\;\frac{1}{2}\;[x+c+\surd \left(x^{2}+2\mathrm{cx}\right)$ [56], where c = 20.
